# ID 45 - Programa de Tutoria para a Elaboração de Protocolos Clínicos e Diretrizes Terapêuticas: resultados da qualidade metodológica e de relato

**DOI:** 10.5327/2237-9622.2025.v34s1.45

**Published:** 2025-11-25

**Authors:** Roseana Boek Carvalho, Roseana Boek Carvalho, Marina Petrasi Guahnon, Muriel Primon de Barros, Bruna Marmett, Ana Paula Blankenheim, Bárbara Cristiane da Silva, Rodrigo Pereira de Almeida, Gilson Pires Dorneles, Cintia Pereira de Araujo, Debora Gräf, Karlyse Belli, Verônica Colpani, Avila Vidal, Brigida Fernandes, Clementina Corah Prado, Cynthia Andrade, Marta Maior, Meline Kron, Thais Melo, Cinara Stein, Suena Medeiros Parahiba, Maicon Falavigna

**Keywords:** protocolos clínicos, Avaliação de Tecnologias em Saúde, assistência à saúde

## Abstract

**Introdução:**

A elaboração de Protocolos Clínicos e Diretrizes Terapêuticas (PCDT) tem o desafio de harmonizar rigor metodológico e padronização de processos, visando garantir a confiabilidade e a reprodutibilidade das recomendações no contexto do Sistema Único de Saúde (SUS). O desenvolvimento de recomendações em saúde embasadas em uma síntese de evidências com rigor metodológico fornece suporte científico essencial para a tomada de decisão dos profissionais e gestores de saúde. As ações de tutorias de Núcleos de Avaliação de Tecnologias em Saúde (Nats) experientes no desenvolvimento de PCDT podem contribuir na padronização de processos no desenvolvimento desses documentos com Nats iniciantes nesse processo. O objetivo deste trabalho foi relatar as ações de tutorias que apoiam os Nats para a elaboração de PCDT para o SUS, bem como descrever a qualidade metodológica e de relato de protocolos já publicados.

**Método:**

O planejamento e a estrutura das tutorias foram elaborados de acordo com as diretrizes metodológicas do Ministério da Saúde (MS). O processo foi definido em encontros virtuais e presenciais. Os Nats participantes, sem experiência prévia no processo de elaboração de PCDT, foram contratados para o desenvolvimento desses documentos junto ao acompanhamento de uma Nats experiente. A qualidade de PCDT publicados pelo MS e desenvolvidos durante a tutoria (2021-2023), foi avaliada por meio da ferramenta AGREE-2 e da lista de verificação RIGHT por pesquisadores independentes que não participaram do processo de tutoria. Os dados descritivos foram apresentados em frequências e percentuais.

**Resultados:**

O programa de tutoria auxiliou 60 pesquisadores de dez Nats na elaboração ou atualização de 16 PCDT, abrangendo as áreas de psiquiatria (2), neurologia (4), oncologia (3), hematologia (2), nefrologia (1), ortopedia e traumatologia (1), endocrinologia (1), doenças infecciosas (1) e gastroenterologia (1). Participaram do processo de desenvolvimento das diretrizes 12 sociedades médicas. No total, foram formuladas 86 perguntas PICO, com foco predominante em questões de tratamento clínico (80). Dos 16 PCDT tutorados, seis foram publicados e avaliados. A aplicação da ferramenta AGREE-2 apontou alta qualidade das diretrizes em "Rigor do desenvolvimento" (92,5%), "Clareza da apresentação" (97,5%) e "Independência editorial" (94,4%), mas menor qualidade em "Aplicabilidade" (73,1%). A qualidade geral das diretrizes foi considerada satisfatória (90%). A verificação com a lista RIGHT indicou um relato de alta qualidade em relação ao contexto, síntese de evidências, recomendações, revisão e garantia de qualidade, além de declaração de financiamento e gestão de conflitos de interesse; no entanto, a clareza da apresentação de informações básicas de cada diretriz ainda necessitam de melhorias.

**Conclusão:**

Os resultados obtidos utilizando ambas ferramentas foram semelhantes em termos de completude dos PCDT, demonstrando que um programa de tutoria de elaboração de PCDT pode ajudar no melhor aperfeiçoamento metodológico durante o desenvolvimento de diretrizes e tornar as recomendações mais sistematizadas, transparentes e compreensíveis. Tendo em vista que o conhecimento compartilhado pode promover o crescimento coletivo e fortalecer a base para práticas de saúde mais efetivas.

